# Editorial: The genome’s rising star: Transfer RNA-derived small fragments

**DOI:** 10.3389/fmolb.2022.979043

**Published:** 2022-08-17

**Authors:** Wei Yan

**Affiliations:** College of Life Sciences, TaiKang Center for Life and Medical Sciences, TaiKang Medical School, Wuhan University, Wuhan, China

**Keywords:** tRNA derived fragments (tRFs), cancer, mitochondria dysfunction, virus infection, translation

In this Research Topic, Choi et al. identified an enzyme “ELAC2” which is responsible for the induction of a functional tRF5 derived from tRNA-Gln-CTG (tRF5-GlnCTG). Interestingly, tRF5-GlnCTG could promote respiratory syncytial virus (RSV) replication and induction. Moreover, they found ELAC2 was associated with RSV N and NS1 proteins. Their findings provide new insights into therapeutic strategy development against RSV infection.

Besides one research article, there are three review papers also included. Bian et al. summarized how tRNAs and tRNA metabolism–associated enzymes play an important role in the occurrence and development of lung cancer beyond translation. Their review covered several different aspects of tRNA metabolism in lung cancer, such as tRNA transcription and mutation, tRNA molecules and derivatives, tRNA-modifying enzymes, and aminoacyl-tRNA synthetases (ARSs), aiming at a better understanding of the pathogenesis of lung cancer and providing new therapeutic strategies for it.

Meseguer overviewed the recent findings of two classes of small non-coding RNAs (sncRNAs): miRNAs and tRFs. They are acting as key elements in the mitochondria–nucleus crosstalk. His review highlights the emerging roles and the interrelation of these sncRNAs in different signaling pathways between mitochondria and the host cell. Their alternation leads to diseases associated with mitochondrial dysfunction.

The fundamental roles of tRFs are emerging in all organisms from mammalians to the world of botany. Alves and Nogueira summarized the most recent development of tRFs in plants, which covered distinctive regulatory layers including transcription and translation regulation, RNA degradation, ribosome biogenesis, stress response, regulatory signaling in plant nodulation, and genome protection against transposable elements.

Conclusively, the biogenesis mechanism and downstream biological function of tRNA-derived fragments are still less defined yet. We believe more and more researchers should get involved in this field and extend its potential clinical applications in the near future.

